# Supplementary material for: Effectiveness of Biofeedback in Individuals with Awake Bruxism Compared to Other Types of Treatment: A Systematic Review
Source: Int J Environ Res Public Health. 2023 Jan 14;20(2):1558. doi: 10.3390/ijerph20021558 (PMC9863342; doi:10.3390/ijerph20021558)
Supplement: Supplementary file 1 [file ijerph-20-01558-s001.zip › ijerph-2064492-supplementary.pdf]

Supplementary Materials

Detail search strategy: OVID/Medline; Embase; PubMed, Cochrane Library, Web of science

- **Ovid MEDLINE(R) ALL 1946 to November 10, 2020**

Number of results: 942

Search date: November 10, 2020

1. exp tooth diseases/ or exp bruxism/ or sleep bruxism/
2. awake bruxism.mp.
3. 1 or 2
4. musculoskeletal manipulations/ or manipulation, chiropractic/ or manipulation, osteopathic/ or therapy, soft tissue/
5. orthopedic.mp.
6. exp musculoskeletal manipulations/ or exp manipulation, orthopedic/ or exp manipulation, osteopathic/ or manipulation, spinal/ or exp therapy, soft tissue/ or myofunctional therapy/
7. exp physical therapy modalities/ or exp dry needling/
8. manual therapy.mp.
9. exp Relaxation Therapy/
10. exp drainage/ or exp drainage, postural/ or exp manual lymphatic drainage/
11. exp spine/ or exp joints/
12. exp Back/
13. exp acupressure/ or exp massage/
14. exp Motion Therapy, Continuous Passive/
15. mulligan.mp.

16. 4 or 5 or 6 or 7 or 8 or 9 or 10 or 11 or 12 or 13 or 14 or 15

17. physical therapy modalities/ or exercise movement techniques/ or exercise therapy/ or hydrotherapy/ or exp rehabilitation/

18. exp exercise/ or exp muscle stretching exercises/ or exp running/ or exp swimming/ or exp walking/ or exp muscle strength/

19. exp weight lifting/ or exp relaxation/

20. (physical and rehabilitation medicine).mp. [mp=title, abstract, original title, name of substance word, subject heading word, floating sub-heading word, keyword heading word, organism supplementary concept word, protocol supplementary concept word, rare disease supplementary concept word, unique identifier, synonyms]

21. exp Posture/th [Therapy]

22. exp Yoga/th [Therapy]

23. pilates.mp.

24. resistance activity.mp.

25. weight training.mp.

26. exp endurance training/ or exp resistance training/

27. strength training.mp.

28. progressive resistance.mp.

29. gravity resistive.mp.

30. exp muscle contraction/ or exp isometric contraction/ or exp isotonic contraction/

31. eccentric contraction.mp.

32. concentric contraction.mp.

33. training program.mp.

34. training protocol.mp.

35. training regime.mp.

36. training strategy.mp.

37. training therapy.mp.

38. training intervention.mp.

39 training progressive.mp.

40. training functional.mp

41 training home.mp.

42. training outpatient.mp.

43. 17 or 18 or 19 or 20 or 21 or 22 or 23 or 24 or 25 or 26 or 27 or 28 or 29 or 30 or 31 or 32 or 33 or 34 or 35 or 36 or 37 or 38 or 39 or 40 or 41 or 42

44. ultrasound.mp.

45. exp electric stimulation therapy/ or exp transcutaneous electric nerve stimulation/ or exp laser therapy/ or exp phototherapy/

46. exp cryotherapy/ or exp electric stimulation therapy/ or exp electroacupuncture/ or exp pulsed radiofrequency treatment/

47. interferential current.mp.

48. exp short-wave therapy/ or exp ultrasonic therapy/

49. exp Electromagnetic Fields/st [Standards]

50. thermotherapy.mp.

51. exp iontophoresis/ or exp phonophoresis/

52. exp Electrophoresis/tu [Therapeutic Use]

53. photobiomodulation.mp.

54. burst-modulated alternating current.mp.

55. russian current.mp.

56. microcurrents.mp.

57. Electrotherapy.mp.

58. 44 or 45 or 46 or 47 or 48 or 49 or 50 or 51 or 52 or 53 or 54 or 55 or 56 or 57

59. exp acupuncture therapy/ or exp acupuncture analgesia/ or exp acupuncture, ear/ or exp moxibustion/ or exp auriculotherapy/ or exp cupping therapy/

60. laser acupuncture.mp.

61. 59 or 60

62. exp laser therapy/ or exp low-level light therapy/

63. low energy laser therapy.mp.

64. low intensity laser therapy.mp.

65. infrared laser.mp.

66. diode laser.mp.

67. soft laser.mp.

68. 62 or 63 or 64 or 65 or 66 or 67

69. exp Diathermy/tu, th [Therapeutic Use, Therapy]

70. exp biofeedback, psychology/ or exp neurofeedback/

71. brainwave biofeedback.mp.

72. electromyography biofeedback.mp.

73. 70 or 71 or 72 11388 Advanced More

74. exp cognitive behavioral therapy/ or exp "acceptance and commitment therapy"/ or exp mindfulness/

75. 16 or 43 or 58 or 61 or 68 or 69 or 73 or 74

76. 3 and 75 77. exp clinical trial/ or exp controlled clinical trial/

78. exp randomized controlled trial/

79. randomly.mp.

80. 77 or 78 or 79

81. 76 and 80

- **Embase 1974 to 2020 November 10, 2020 (OVID interface)**

Number of results: 328

Search date: November 10,2020

1. (bruxism 'sleep bruxism' 'awake bruxism' manipulation orthopedic chiropractic 'spinal manipulation' 'spinal adjustment' 'musculoskeletal therapy' 'musculoskeletal manipulations' 'manual therapy' 'physical therapy modalities' 'relaxation therapy' 'orthopedic procedures' 'osteopathic medicine' 'drainage' 'dry needling' 'myofunctional therapy' spine spinal back osteopathic massage masseuse 'passive jaw motion device' 'continuous passive motion' mulligan ultrasound 'transcutaneous electrical stimulation' 'electrostimulation' 'interferential current' biofeedback laser shortwave 'electromagnetic fields' heat cold iontophoresis electrophoresis photobiomodulation phototherapy 'burst modulated alternating current' 'russian current' 'microcurrents' 'electrotherapy' 'interferential therapy' 'physical therapy' 'exercise therapy' 'exercise movement techniques' 'exercise' 'muscle stretching exercises' 'weight lifting' 'physical and rehabilitation medicine' 'rehabilitation' posture 'relaxation training' yoga pilates 'resistance activity' 'weight training' 'resistance training' 'strength training' 'muscle strengthening' 'weightlifting' 'progressive resistance' 'gravity resistive' 'isotonic contraction' 'isometric contraction' 'eccentric' 'concentric contraction' 'training program' 'training protocol' 'training regime' 'training strategy' 'training therapy' 'training intervention' 'training progressive' 'training functional' 'training home' 'training home-based' 'training outpatient' electroacupuncture acupressure 'laser acupuncture' 'auricular acupuncture' 'acupuncture' 'medical acupuncture' 'laser therapy' 'low level laser therapy' 'low intensity laser therapy' 'low energy laser therapy' 'infrared laser' 'ir laser' 'diode laser' 'soft laser' 'transcutaneous electric nerve stimulation' 'pulsed radio frequency energy' 'diathermy' neurofeedback 'brainwave biofeedback' 'electromyography feedback' 'biofeedback' 'cognitive behavioral therapy' 'cognitive therapy' 'acceptance and commitment therapy' mindfulness 'behavior therapy' 'clinical trial' 'clinical trial' 'randomized' 'placebo' 'placebo' 'randomly' groups)

- **PubMed 1949 to November 10, 2020**

Number of results: 786

Search date: November 10,2020

1. Bruxism

2. Sleep bruxism

3. Awake bruxism

4. ((bruxism) OR ("sleep bruxism")) OR ("awake bruxism")

5. ((manipulation) OR (Orthopedic) OR (chiropractic) OR ("spinal manipulation") OR ("spinal adjustment") OR ("musculoskeletal therapy") OR ("musculoskeletal manipulations") OR ("manual therapy") OR ("physical therapy modalities") OR ("relaxation therapy") OR ("orthopedic procedures") OR ("osteopathic medicine") OR ("drainage") OR ("dry needling") OR ("myofunctional therapy") OR (spine) OR (spinal) OR (back) OR (osteopathic) OR (massage) OR (masseuse) OR ("passive jaw motion device") OR ("continuous passive motion") OR (mulligan))

6. ((ultrasound) OR ("transcutaneous electrical stimulation") OR ("electrostimulation") OR ("interferential current") OR (biofeedback) OR (laser) OR (shortwave) OR ("electromagnetic fields") OR (heat) OR (cold) OR (iontophoresis) OR (electrophoresis) OR (photobiomodulation) OR (phototherapy) OR (electroacupuncture) OR ("burst modulated alternating current") OR ("russian current") OR ("microcurrents") OR ("electrotherapy") OR ("interferential therapy"))

7. (("physical therapy") OR ("Exercise therapy") OR ("exercise movement techniques") OR ("exercise") OR ("muscle stretching exercises") OR ("weight lifting") OR ("physical and rehabilitation medicine") OR ("rehabilitation") OR (posture) OR ("relaxation training") OR (yoga) OR (Pilates) OR ("resistance activity") OR ("weight training") OR ("resistance training") OR ("strength training") OR ("muscle strengthening") OR ("weightlifting") OR ("progressive resistance") OR ("gravity resistive") OR ("isotonic contraction") OR ("isometric contraction") OR ("eccentric") OR ("concentric contraction") OR ("training program") OR ("training protocol") OR ("training regime") OR ("training strategy") OR ("training therapy") OR ("training

intervention") OR ("training progressive") OR ("training functional") OR ("training home") OR ("training home-based") OR ("training outpatient"))

8. ((electroacupuncture) OR (acupressure) OR ("laser acupuncture") OR ("auricular acupuncture") OR ("acupuncture") OR ("medical acupuncture"))

9. (("laser therapy") OR ("low level laser therapy") OR ("low intensity laser therapy") OR ("low energy laser therapy") OR ("infrared laser") OR ("IR laser") OR ("diode laser") OR ("soft laser"))

10. (("transcutaneous electric nerve stimulation") OR ("pulsed radio frequency energy") OR ("diathermy"))

11. ((neurofeedback) OR ("brainwave biofeedback") OR ("electromyography feedback") OR ("biofeedback"))

12. (("cognitive behavioral therapy") OR ("cognitive therapy") OR ("acceptance and commitment therapy") OR (mindfulness) OR ("behavior therapy"))

13. (("clinical trial") OR ("randomized") OR ("placebo") OR ("randomly") OR (groups))

14. ((((((((manipulation) OR (Orthopedic) OR (chiropractic) OR ("spinal manipulation") OR ("spinal adjustment") OR ("musculoskeletal therapy") OR ("musculoskeletal manipulations") OR ("manual therapy") OR ("physical therapy modalities") OR ("relaxation therapy") OR ("orthopedic procedures") OR ("osteopathic medicine") OR ("drainage") OR ("dry needling") OR ("myofunctional therapy") OR (spine) OR (spinal) OR (back) OR (osteopathic) OR (massage) OR (masseuse) OR ("passive jaw motion device") OR ("continuous passive motion") OR (mulligan))) OR (((ultrasound) OR ("transcutaneous electrical stimulation") OR ("electrostimulation") OR ("interferential current") OR (biofeedback) OR (laser) OR (shortwave) OR ("electromagnetic fields") OR (heat) OR (cold) OR (iontophoresis) OR (electrophoresis) OR (photobiomodulation) OR (phototherapy) OR (electroacupuncture) OR ("burst modulated alternating current") OR ("russian current") OR ("microcurrents") OR ("electrotherapy") OR ("interferential therapy")))) OR ((("physical therapy") OR ("Exercise therapy") OR ("exercise movement techniques") OR ("exercise") OR ("muscle stretching exercises") OR ("weight lifting") OR ("physical and rehabilitation medicine") OR ("rehabilitation") OR (posture) OR ("relaxation training") OR (yoga)

OR (Pilates) OR ("resistance activity") OR ("weight training") OR ("resistance training") OR ("strength training") OR ("muscle strengthening") OR ("weightlifting") OR ("progressive resistance") OR ("gravity resistive") OR ("isotonic contraction") OR ("isometric contraction") OR ("eccentric") OR ("concentric contraction") OR ("training program") OR ("training protocol") OR ("training regime") OR ("training strategy") OR ("training therapy") OR ("training intervention") OR ("training progressive") OR ("training functional") OR ("training home") OR ("training home-based") OR ("training outpatient")))) OR (((electroacupuncture) OR (acupressure) OR ("laser acupuncture") OR ("auricular acupuncture") OR ("acupuncture") OR ("medical acupuncture")))) OR ((("laser therapy") OR ("low level laser therapy") OR ("low intensity laser therapy") OR ("low energy laser therapy") OR ("infrared laser") OR ("IR laser") OR ("diode laser") OR ("soft laser")))) OR ((("transcutaneous electric nerve stimulation") OR ("pulsed radio frequency energy") OR ("diathermy")))) OR (((neurofeedback) OR ("brainwave biofeedback") OR ("electromyography feedback") OR ("biofeedback")))) OR ((("cognitive behavioral therapy") OR ("cognitive therapy") OR ("acceptance and commitment therapy") OR (mindfulness) OR ("behavior therapy")))

15. (((((((((manipulation) OR (Orthopedic) OR (chiropractic) OR ("spinal manipulation") OR ("spinal adjustment") OR ("musculoskeletal therapy") OR ("musculoskeletal manipulations") OR ("manual therapy") OR ("physical therapy modalities") OR ("relaxation therapy") OR ("orthopedic procedures") OR ("osteopathic medicine") OR ("drainage") OR ("dry needling") OR ("myofunctional therapy") OR (spine) OR (spinal) OR (back) OR (osteopathic) OR (massage) OR (masseuse) OR ("passive jaw motion device") OR ("continuous passive motion") OR (mulligan))) OR (((ultrasound) OR ("transcutaneous electrical stimulation") OR ("electrostimulation") OR ("interferential current") OR (biofeedback) OR (laser) OR (shortwave) OR ("electromagnetic fields") OR (heat) OR (cold) OR (iontophoresis) OR (electrophoresis) OR (photobiomodulation) OR (phototherapy) OR (electroacupuncture) OR ("burst modulated alternating current") OR ("russian current") OR ("microcurrents") OR ("electrotherapy") OR ("interferential therapy")))) OR ((("physical therapy") OR ("Exercise therapy") OR ("exercise movement techniques") OR ("exercise") OR ("muscle stretching exercises") OR ("weight lifting") OR ("physical and rehabilitation medicine") OR ("rehabilitation") OR (posture) OR ("relaxation training") OR (yoga) OR (Pilates) OR ("resistance activity") OR ("weight training") OR ("resistance

training") OR ("strength training") OR ("muscle strengthening") OR ("weightlifting") OR ("progressive resistance") OR ("gravity resistive") OR ("isotonic contraction") OR ("isometric contraction") OR ("eccentric") OR ("concentric contraction") OR ("training program") OR ("training protocol") OR ("training regime") OR ("training strategy") OR ("training therapy") OR ("training intervention") OR ("training progressive") OR ("training functional") OR ("training home") OR ("training home-based") OR ("training outpatient")))) OR (((electroacupuncture) OR (acupressure) OR ("laser acupuncture") OR ("auricular acupuncture") OR ("acupuncture") OR ("medical acupuncture")))) OR ((("laser therapy") OR ("low level laser therapy") OR ("low intensity laser therapy") OR ("low energy laser therapy") OR ("infrared laser") OR ("IR laser") OR ("diode laser") OR ("soft laser")))) OR ((("transcutaneous electric nerve stimulation") OR ("pulsed radio frequency energy") OR ("diathermy")))) OR (((neurofeedback) OR ("brainwave biofeedback") OR ("electromyography feedback") OR ("biofeedback")))) OR ((("cognitive behavioral therapy") OR ("cognitive therapy") OR ("acceptance and commitment therapy") OR (mindfulness) OR ("behavior therapy")))) AND ((("clinical trial") OR ("randomized") OR ("placebo") OR ("randomly") OR (groups)))

16. (((bruxism) OR ("sleep bruxism")) OR ("awake bruxism")) AND ((((((((((manipulation) OR (Orthopedic) OR (chiropractic) OR ("spinal manipulation") OR ("spinal adjustment") OR ("musculoskeletal therapy") OR ("musculoskeletal manipulations") OR ("manual therapy") OR ("physical therapy modalities") OR ("relaxation therapy") OR ("orthopedic procedures") OR ("osteopathic medicine") OR ("drainage") OR ("dry needling") OR ("myofunctional therapy") OR (spine) OR (spinal) OR (back) OR (osteopathic) OR (massage) OR (masseuse) OR ("passive jaw motion device") OR ("continuous passive motion") OR (mulligan))) OR (((ultrasound) OR ("transcutaneous electrical stimulation") OR ("electrostimulation") OR ("interferential current") OR (biofeedback) OR (laser) OR (shortwave) OR ("electromagnetic fields") OR (heat) OR (cold) OR (iontophoresis) OR (electrophoresis) OR (photobiomodulation) OR (phototherapy) OR (electroacupuncture) OR ("burst modulated alternating current") OR ("russian current") OR ("microcurrents") OR ("electrotherapy") OR ("interferential therapy")))) OR ((("physical therapy") OR ("Exercise therapy") OR ("exercise movement techniques") OR ("exercise") OR ("muscle stretching exercises") OR ("weight lifting") OR ("physical and rehabilitation medicine") OR ("rehabilitation") OR (posture) OR ("relaxation training") OR (yoga)

OR (Pilates) OR ("resistance activity") OR ("weight training") OR ("resistance training") OR ("strength training") OR ("muscle strengthening") OR ("weightlifting") OR ("progressive resistance") OR ("gravity resistive") OR ("isotonic contraction") OR ("isometric contraction") OR ("eccentric") OR ("concentric contraction") OR ("training program") OR ("training protocol") OR ("training regime") OR ("training strategy") OR ("training therapy") OR ("training intervention") OR ("training progressive") OR ("training functional") OR ("training home") OR ("training home-based") OR ("training outpatient")))) OR (((electroacupuncture) OR (acupressure) OR ("laser acupuncture") OR ("auricular acupuncture") OR ("acupuncture") OR ("medical acupuncture")))) OR ((("laser therapy") OR ("low level laser therapy") OR ("low intensity laser therapy") OR ("low energy laser therapy") OR ("infrared laser") OR ("IR laser") OR ("diode laser") OR ("soft laser")))) OR ((("transcutaneous electric nerve stimulation") OR ("pulsed radio frequency energy") OR ("diathermy")))) OR (((neurofeedback) OR ("brainwave biofeedback") OR ("electromyography feedback") OR ("biofeedback")))) OR ((("cognitive behavioral therapy") OR ("cognitive therapy") OR ("acceptance and commitment therapy") OR (mindfulness) OR ("behavior therapy")))) AND ((("clinical trial") OR ("randomized") OR ("placebo") OR ("randomly") OR (groups))))

17. (((bruxism) OR ("sleep bruxism")) OR ("awake bruxism")) AND (((((((((manipulation) OR (Orthopedic) OR (chiropractic) OR ("spinal manipulation") OR ("spinal adjustment") OR ("musculoskeletal therapy") OR ("musculoskeletal manipulations") OR ("manual therapy") OR ("physical therapy modalities") OR ("relaxation therapy") OR ("orthopedic procedures") OR ("osteopathic medicine") OR ("drainage") OR ("dry needling") OR ("myofunctional therapy") OR (spine) OR (spinal) OR (back) OR (osteopathic) OR (massage) OR (masseuse) OR ("passive jaw motion device") OR ("continuous passive motion") OR (mulligan))) OR (((ultrasound) OR ("transcutaneous electrical stimulation") OR ("electrostimulation") OR ("interferential current") OR (biofeedback) OR (laser) OR (shortwave) OR ("electromagnetic fields") OR (heat) OR (cold) OR (iontophoresis) OR (electrophoresis) OR (photobiomodulation) OR (phototherapy) OR (electroacupuncture) OR ("burst modulated alternating current") OR ("russian current") OR ("microcurrents") OR ("electrotherapy") OR ("interferential therapy")))) OR ((("physical therapy") OR ("Exercise therapy") OR ("exercise movement techniques") OR ("exercise") OR ("muscle stretching exercises") OR ("weight lifting") OR ("physical and rehabilitation

medicine") OR ("rehabilitation") OR (posture) OR ("relaxation training") OR (yoga) OR (Pilates) OR ("resistance activity") OR ("weight training") OR ("resistance training") OR ("strength training") OR ("muscle strengthening") OR ("weightlifting") OR ("progressive resistance") OR ("gravity resistive") OR ("isotonic contraction") OR ("isometric contraction") OR ("eccentric") OR ("concentric contraction") OR ("training program") OR ("training protocol") OR ("training regime") OR ("training strategy") OR ("training therapy") OR ("training intervention") OR ("training progressive") OR ("training functional") OR ("training home") OR ("training home-based") OR ("training outpatient")))) OR (((electroacupuncture) OR (acupressure) OR ("laser acupuncture") OR ("auricular acupuncture") OR ("acupuncture") OR ("medical acupuncture")))) OR ((("laser therapy") OR ("low level laser therapy") OR ("low intensity laser therapy") OR ("low energy laser therapy") OR ("infrared laser") OR ("IR laser") OR ("diode laser") OR ("soft laser")))) OR ((("transcutaneous electric nerve stimulation") OR ("pulsed radio frequency energy") OR ("diathermy")))) OR (((neurofeedback) OR ("brainwave biofeedback") OR ("electromyography feedback") OR ("biofeedback")))) OR ((("cognitive behavioral therapy") OR ("cognitive therapy") OR ("acceptance and commitment therapy") OR (mindfulness) OR ("behavior therapy"))))

- **Web of Science (Indexes=SCI-EXPANDED, SSCI, A&HCI, ESCI) Timespan: all years**

Number of results: 1.579

Search date: October 28,2020

1. (awake bruxism OR diurnal bruxism OR oral parafunction OR grinding OR clenching OR bruxism)

2. (awake bruxism OR diurnal bruxism OR oral parafunction OR grinding OR clenching OR bruxism) Refined by: WEB OF SCIENCE CATEGORIES: (BIOPHYSICS OR ORTHOPEDICS OR MEDICINE GENERAL INTERNAL OR SURGERY OR PHYSIOLOGY OR DENTISTRY ORAL SURGERY MEDICINE OR PHARMACOLOGY PHARMACY)

3. (acupuncture OR acupuncture therapy OR electroacupuncture OR acupressure OR laser acupuncture OR auricular acupuncture OR medical acupuncture)

4. (acupuncture OR acupuncture therapy OR electroacupuncture OR acupressure OR laser acupuncture OR auricular acupuncture OR medical acupuncture) Refined by: WEB OF SCIENCE CATEGORIES: (BIOPHYSICS OR ORTHOPEDICS OR MEDICINE GENERAL INTERNAL OR SURGERY OR PHYSIOLOGY OR DENTISTRY ORAL SURGERY MEDICINE OR PHARMACOLOGY PHARMACY)

5. (laser therapy OR low level laser therapy OR low intensity laser therapy OR low energy laser therapy OR infrared therapy OR diode laser OR soft laser)

6. (laser therapy OR low level laser therapy OR low intensity laser therapy OR low energy laser therapy OR infrared therapy OR diode laser OR soft laser) Refined by: WEB OF SCIENCE CATEGORIES: (SURGERY OR PATHOLOGY OR CRITICAL CARE MEDICINE OR RHEUMATOLOGY OR PHARMACOLOGY PHARMACY OR REHABILITATION OR HEALTH CARE SCIENCES SERVICES OR DENTISTRY ORAL SURGERY MEDICINE OR ORTHOPEDICS OR MULTIDISCIPLINARY SCIENCES OR MEDICINE GENERAL INTERNAL OR GERIATRICS GERONTOLOGY)

7. (ultrasound OR transcutaneous electrical stimulation OR TENS OR electrostimulation OR interferential current OR IFC OR shortwave OR electromagnetic field OR heat OR cold OR iontophoresis OR electrophoresis OR electroacupuncture OR photobiomodulation OR phototherapy OR microcurrent OR interferential therapy OR burst modulated alternating current)

8. (ultrasound OR transcutaneous electrical stimulation OR TENS OR electrostimulation OR interferential current OR IFC OR shortwave OR electromagnetic field OR heat OR cold OR iontophoresis OR electrophoresis OR electroacupuncture OR photobiomodulation OR phototherapy OR microcurrent OR interferential therapy OR burst modulated alternating current) Refined by: WEB OF SCIENCE CATEGORIES: (PHARMACOLOGY PHARMACY OR MEDICINE GENERAL INTERNAL OR RADIOLOGY NUCLEAR MEDICINE MEDICAL IMAGING OR SURGERY OR PHYSIOLOGY OR MULTIDISCIPLINARY SCIENCES OR ORTHOPEDICS)

9. (electrotherapy OR diathermy OR transcutaneous electric nerve stimulation OR pulsed radio frequency energy)

10. (electrotherapy OR diathermy OR transcutaneous electric nerve stimulation OR pulsed radio frequency energy) Refined by: WEB OF SCIENCE CATEGORIES: (SURGERY OR INTEGRATIVE COMPLEMENTARY MEDICINE OR PRIMARY HEALTH CARE OR MEDICINE GENERAL INTERNAL OR REHABILITATION OR PSYCHIATRY OR PATHOLOGY OR PHYSIOLOGY OR PHARMACOLOGY PHARMACY OR ORTHOPEDICS OR BEHAVIORAL SCIENCES OR NURSING OR HEALTH CARE SCIENCES SERVICES OR DENTISTRY ORAL SURGERY MEDICINE OR MEDICINE RESEARCH EXPERIMENTAL OR CRITICAL CARE MEDICINE OR RHEUMATOLOGY OR PSYCHOLOGY CLINICAL OR MULTIDISCIPLINARY SCIENCES)

11. (biofeedback OR neurofeedback Or brainwave feedback OR electromyography feedback OR electromyography biofeedback device OR electromyography biofeedback training)

12. (biofeedback OR neurofeedback Or brainwave feedback OR electromyography feedback OR electromyography biofeedback device OR electromyography biofeedback training) Refined by: WEB OF SCIENCE CATEGORIES: (NEUROSCIENCES OR HEALTH CARE SCIENCES SERVICES OR EDUCATION EDUCATIONAL RESEARCH OR PSYCHOLOGY CLINICAL OR NURSING OR REHABILITATION OR PSYCHOLOGY APPLIED OR PSYCHIATRY OR LINGUISTICS OR PSYCHOLOGY PSYCHOANALYSIS OR RHEUMATOLOGY OR PSYCHOLOGY OR SURGERY OR CRITICAL CARE MEDICINE OR MEDICINE GENERAL INTERNAL OR FAMILY STUDIES OR PHYSIOLOGY OR PSYCHOLOGY EXPERIMENTAL OR EDUCATION SPECIAL OR RADIOLOGY NUCLEAR MEDICINE MEDICAL IMAGING OR ORTHOPEDICS OR PSYCHOLOGY BIOLOGICAL OR PSYCHOLOGY MULTIDISCIPLINARY OR PSYCHOLOGY EDUCATIONAL OR MULTIDISCIPLINARY SCIENCES OR PRIMARY HEALTH CARE OR BEHAVIORAL SCIENCES OR MEDICINE RESEARCH EXPERIMENTAL OR PSYCHOLOGY DEVELOPMENTAL OR PSYCHOLOGY SOCIAL OR INTEGRATIVE COMPLEMENTARY MEDICINE OR PHARMACOLOGY PHARMACY OR DENTISTRY ORAL SURGERY MEDICINE OR ANESTHESIOLOGY)

13. (education OR cognitive behavioral therapy OR cognitive therapy OR behavior therapy OR mindfulness Or patient education handout)

14. (education OR cognitive behavioral therapy OR cognitive therapy OR behavior therapy OR mindfulness Or patient education handout) Refined by: WEB OF SCIENCE CATEGORIES: (EDUCATION EDUCATIONAL RESEARCH OR PSYCHIATRY OR MEDICINE GENERAL INTERNAL OR CRITICAL CARE MEDICINE OR EDUCATION SCIENTIFIC DISCIPLINES OR DENTISTRY ORAL SURGERY MEDICINE OR NURSING OR BEHAVIORAL SCIENCES OR HEALTH CARE SCIENCES SERVICES OR PSYCHOLOGY CLINICAL OR PSYCHOLOGY APPLIED OR PSYCHOLOGY EXPERIMENTAL OR RHEUMATOLOGY OR NEUROSCIENCES OR ANESTHESIOLOGY OR REHABILITATION OR FAMILY STUDIES OR PSYCHOLOGY MULTIDISCIPLINARY OR PHARMACOLOGY PHARMACY OR SURGERY OR PSYCHOLOGY OR MULTIDISCIPLINARY SCIENCES OR PSYCHOLOGY DEVELOPMENTAL OR PRIMARY HEALTH CARE OR ORTHOPEDICS OR PSYCHOLOGY EDUCATIONAL OR MEDICINE RESEARCH EXPERIMENTAL OR EDUCATION SPECIAL OR PATHOLOGY OR LINGUISTICS)

15. (exercise therapy OR posture training OR movement therapy OR motion therapy OR physiotherapy OR breathing exercise OR muscle stretching exercise OR rehabilitation exercise OR myotherapy OR orofacial myotherapy OR oral therapy OR myofunctional therapy OR physical activity OR physical exercise OR isometric exercise OR acute exercise OR training OR exercise training OR physical education training OR training program OR exercise movement technique)

16. (exercise therapy OR posture training OR movement therapy OR motion therapy OR physiotherapy OR breathing exercise OR muscle stretching exercise OR rehabilitation exercise OR myotherapy OR orofacial myotherapy OR oral therapy OR myofunctional therapy OR physical activity OR physical exercise OR isometric exercise OR acute exercise OR training OR exercise training OR physical education training OR training program OR exercise movement technique) Refined by: WEB OF SCIENCE CATEGORIES: (MEDICINE GENERAL INTERNAL OR NEUROSCIENCES OR BEHAVIORAL SCIENCES OR PSYCHOLOGY DEVELOPMENTAL OR REHABILITATION OR DENTISTRY ORAL SURGERY MEDICINE OR PHARMACOLOGY PHARMACY OR PSYCHOLOGY APPLIED

OR HEALTH POLICY SERVICES OR SURGERY OR EDUCATION EDUCATIONAL RESEARCH OR PSYCHIATRY OR RHEUMATOLOGY OR PSYCHOLOGY EXPERIMENTAL OR PRIMARY HEALTH CARE OR PHYSIOLOGY OR HEALTH CARE SCIENCES SERVICES OR MULTIDISCIPLINARY SCIENCES OR ORTHOPEDICS OR NURSING OR CRITICAL CARE MEDICINE OR MEDICINE RESEARCH EXPERIMENTAL OR PSYCHOLOGY MULTIDISCIPLINARY OR PSYCHOLOGY OR ANESTHESIOLOGY OR PSYCHOLOGY CLINICAL OR EMERGENCY MEDICINE OR EDUCATION SCIENTIFIC DISCIPLINES)

17. (orthopedic manipulation OR chiropractic OR spinal adjustment OR osteopathic medicine OR musculoskeletal manipulation OR musculoskeletal therapy OR manual therapy OR physical therapy modalities OR rehabilitation therapy OR relaxation therapy OR relaxation training OR physiotherapy)

18. (orthopedic manipulation OR chiropractic OR spinal adjustment OR osteopathic medicine OR musculoskeletal manipulation OR musculoskeletal therapy OR manual therapy OR physical therapy modalities OR rehabilitation therapy OR relaxation therapy OR relaxation training OR physiotherapy) Refined by: WEB OF SCIENCE CATEGORIES: (REHABILITATION OR HEALTH POLICY SERVICES OR MEDICINE GENERAL INTERNAL OR PSYCHOLOGY MULTIDISCIPLINARY OR ORTHOPEDICS OR NEUROSCIENCES OR PSYCHOLOGY EXPERIMENTAL OR SURGERY OR PSYCHIATRY OR HEALTH CARE SCIENCES SERVICES OR PSYCHOLOGY APPLIED OR INTEGRATIVE COMPLEMENTARY MEDICINE OR EDUCATION SCIENTIFIC DISCIPLINES OR PATHOLOGY OR PSYCHOLOGY CLINICAL OR RHEUMATOLOGY OR EMERGENCY MEDICINE OR PRIMARY HEALTH CARE OR PHARMACOLOGY PHARMACY OR MEDICINE RESEARCH EXPERIMENTAL OR FAMILY STUDIES OR ANESTHESIOLOGY OR CRITICAL CARE MEDICINE OR NURSING OR DENTISTRY ORAL SURGERY MEDICINE OR PSYCHOLOGY DEVELOPMENTAL OR PSYCHOLOGY OR EDUCATION EDUCATIONAL RESEARCH OR EDUCATION SPECIAL OR BEHAVIORAL SCIENCES OR PHYSIOLOGY OR MULTIDISCIPLINARY SCIENCES OR PSYCHOLOGY PSYCHOANALYSIS OR OTORHINOLARYNGOLOGY)

19. #18 OR #16 OR #14 OR #12 OR #10 OR #8 OR #6 OR #4

20. #18 OR #16 OR #14 OR #12 OR #10 OR #8 OR #6 OR #4 Refined by: WEB OF SCIENCE CATEGORIES: (PATHOLOGY OR SURGERY OR PSYCHOLOGY EDUCATIONAL OR PHARMACOLOGY PHARMACY OR PRIMARY HEALTH CARE OR PSYCHIATRY OR MULTIDISCIPLINARY SCIENCES OR REHABILITATION OR HEALTH CARE SCIENCES SERVICES OR FAMILY STUDIES OR PHYSIOLOGY OR EDUCATION SCIENTIFIC DISCIPLINES OR PSYCHOLOGY CLINICAL OR PSYCHOLOGY MULTIDISCIPLINARY OR ORTHOPEDICS OR MEDICINE RESEARCH EXPERIMENTAL OR PSYCHOLOGY OR DENTISTRY ORAL SURGERY MEDICINE OR PSYCHOLOGY APPLIED OR BEHAVIORAL SCIENCES OR INTEGRATIVE COMPLEMENTARY MEDICINE OR PSYCHOLOGY DEVELOPMENTAL OR EDUCATION SPECIAL)

21. #19 AND #2

22. #19 AND #2 Refined by: WEB OF SCIENCE CATEGORIES: (PHYSIOLOGY OR ORTHOPEDICS OR DENTISTRY ORAL SURGERY MEDICINE OR HEALTH CARE SCIENCES SERVICES OR SURGERY OR NEUROSCIENCES OR PSYCHIATRY OR PSYCHOLOGY EXPERIMENTAL OR PHARMACOLOGY PHARMACY OR PSYCHOLOGY MULTIDISCIPLINARY OR REHABILITATION OR EDUCATION SCIENTIFIC DISCIPLINES OR CLINICAL NEUROLOGY OR BEHAVIORAL SCIENCES OR MEDICINE RESEARCH EXPERIMENTAL OR PSYCHOLOGY OR PRIMARY HEALTH CARE OR PATHOLOGY OR MULTIDISCIPLINARY SCIENCES OR CRITICAL CARE MEDICINE)

23. #19 AND #2 Refined by: WEB OF SCIENCE CATEGORIES: (PHYSIOLOGY OR ORTHOPEDICS OR DENTISTRY ORAL SURGERY MEDICINE OR HEALTH CARE SCIENCES SERVICES OR SURGERY OR NEUROSCIENCES OR PSYCHIATRY OR PSYCHOLOGY EXPERIMENTAL OR PHARMACOLOGY PHARMACY OR PSYCHOLOGY MULTIDISCIPLINARY OR REHABILITATION OR EDUCATION SCIENTIFIC DISCIPLINES OR CLINICAL NEUROLOGY OR BEHAVIORAL SCIENCES OR MEDICINE RESEARCH EXPERIMENTAL OR PSYCHOLOGY OR PRIMARY HEALTH CARE OR PATHOLOGY OR MULTIDISCIPLINARY SCIENCES OR CRITICAL CARE MEDICINE) AND WEB OF SCIENCE CATEGORIES: (PHYSIOLOGY OR ORTHOPEDICS OR DENTISTRY ORAL SURGERY MEDICINE OR SURGERY OR HEALTH CARE

SCIENCES SERVICES OR PHARMACOLOGY PHARMACY OR PSYCHIATRY OR PSYCHOLOGY EXPERIMENTAL OR REHABILITATION OR PSYCHOLOGY MULTIDISCIPLINARY OR EDUCATION SCIENTIFIC DISCIPLINES OR MEDICINE RESEARCH EXPERIMENTAL OR BEHAVIORAL SCIENCES OR PSYCHOLOGY OR PRIMARY HEALTH CARE OR MULTIDISCIPLINARY SCIENCES)

- **Cochrane Library (Cochrane Database of Systematic Reviews and Cochrane CENTRAL Register of Controlled trials)**

Numbers of Results: 148

Date searched: Nov 3, 2020

1. awake bruxism OR sleep bruxism OR bruxism

2. manipulation OR Orthopedic OR chiropractic OR spinal manipulation OR spinal adjustment OR musculoskeletal therapy OR musculoskeletal manipulations OR manual therapy OR physical therapy modalities OR relaxation therapy OR orthopedic procedures OR osteopathic medicine OR drainage OR dry needling OR myofunctional therapy OR spine OR spinal OR back OR osteopathic OR massage OR masseuse OR passive jaw motion device OR continuous passive motion OR mulligan

3. physical therapy OR Exercise therapy OR exercise movement techniques OR exercise OR muscle stretching exercises OR weight lifting OR physical and rehabilitation medicine OR rehabilitation OR posture OR relaxation training OR yoga OR pilates OR resistance activity OR weight training OR resistance training OR strength training OR muscle strengthening OR weightlifting OR progressive resistance OR gravity resistive OR isotonic contraction OR isometric contraction OR eccentric OR concentric contraction OR training program OR training protocol OR training regime OR training strategy OR training therapy OR training intervention OR training progressive OR training functional OR training home OR training home-based OR training outpatient

4. ultrasound OR transcutaneous electrical stimulation OR electrostimulation OR interferential current OR biofeedback OR laser OR shortwave OR electromagnetic fields OR heat OR cold OR iontophoresis OR electrophoresis OR photo biomodulation

OR phototherapy OR electroacupuncture OR burst modulated alternating current OR russian current OR microcurrents OR electrotherapy OR inferential therapy

5. electroacupuncture OR acupressure OR acupressure OR laser acupuncture OR auricular acupuncture OR acupuncture OR medical acupuncture

6. laser therapy OR low level laser therapy OR low intensity laser therapy OR low energy laser therapy OR infrared laser OR IR laser OR diode laser OR soft laser

7. transcutaneous electric nerve stimulation OR pulsed radio frequency energy OR diathermy

8. exp clinical trial OR randomized.tw. OR placebo OR randomly.tw. OR groups.tw.

9. neurobiofeedback OR brainwave biofeedback OR electromyography feedback OR biofeedback

10. cognitive behavioral therapy OR cognitive therapy OR acceptance and commitment therapy OR mindfulness OR behaviour therapy

11. #2 OR #3 OR #4 OR #5 OR #6 OR #7 OR #9 OR #10

12. #11 AND #8

13. #1 AND #12

14. #1 AND #11

Uptade: New searches – July 12, 2022

- **PubMed**
  Number of results: 6
  Date searched: July 12, 2022

(((bruxism) OR ("sleep bruxism")) OR ("awake bruxism")) AND (((((((((manipulation) OR (Orthopedic) OR (chiropractic) OR ("spinal manipulation") OR ("spinal adjustment") OR ("musculoskeletal therapy") OR ("musculoskeletal manipulations") OR ("manual therapy") OR ("physical therapy modalities") OR ("relaxation therapy")

OR ("orthopedic procedures") OR ("osteopathic medicine") OR ("drainage") OR ("dry needling") OR ("myofunctional therapy") OR (spine) OR (spinal) OR (back) OR (osteopathic) OR (massage) OR (masseuse) OR ("passive jaw motion device") OR ("continuous passive motion") OR (mulligan))) OR (((ultrasound) OR ("transcutaneous electrical stimulation") OR ("electrostimulation") OR ("interferential current") OR (biofeedback) OR (laser) OR (shortwave) OR ("electromagnetic fields") OR (heat) OR (cold) OR (iontophoresis) OR (electrophoresis) OR (photobiomodulation) OR (phototherapy) OR (electroacupuncture) OR ("burst modulated alternating current") OR ("russian current") OR ("microcurrents") OR ("electrotherapy") OR ("interferential therapy")))) OR ((("physical therapy") OR ("Exercise therapy") OR ("exercise movement techniques") OR ("exercise") OR ("muscle stretching exercises") OR ("weight lifting") OR ("physical and rehabilitation medicine") OR ("rehabilitation") OR (posture) OR ("relaxation training") OR (yoga) OR (Pilates) OR ("resistance activity") OR ("weight training") OR ("resistance training") OR ("strength training") OR ("muscle strengthening") OR ("weightlifting") OR ("progressive resistance") OR ("gravity resistive") OR ("isotonic contraction") OR ("isometric contraction") OR ("eccentric") OR ("concentric contraction") OR ("training program") OR ("training protocol") OR ("training regime") OR ("training strategy") OR ("training therapy") OR ("training intervention") OR ("training progressive") OR ("training functional") OR ("training home") OR ("training home-based") OR ("training outpatient")))) OR (((electroacupuncture) OR (acupressure) OR ("laser acupuncture") OR ("auricular acupuncture") OR ("acupuncture") OR ("medical acupuncture")))) OR ((("laser therapy") OR ("low level laser therapy") OR ("low intensity laser therapy") OR ("low energy laser therapy") OR ("infrared laser") OR ("IR laser") OR ("diode laser") OR ("soft laser")))) OR ((("transcutaneous electric nerve stimulation") OR ("pulsed radio frequency energy") OR ("diathermy")))) OR (((neurofeedback) OR ("brainwave biofeedback") OR ("electromyography feedback") OR ("biofeedback")))) OR ((("cognitive behavioral therapy") OR ("cognitive therapy") OR ("acceptance and commitment therapy") OR (mindfulness) OR ("behavior therapy"))))

- **Cochrane Library (Cochrane Database of Systematic Reviews and Cochrane CENTRAL Register of Controlled trials)**

Numbers of Results: 14

Date searched: July 12, 2022

1. awake bruxism OR sleep bruxism OR bruxism

2. manipulation OR Orthopedic OR chiropractic OR spinal manipulation OR spinal adjustment OR musculoskeletal therapy OR musculoskeletal manipulations OR manual therapy OR physical therapy modalities OR relaxation therapy OR orthopedic procedures OR osteopathic medicine OR drainage OR dry needling OR myofunctional therapy OR spine OR spinal OR back OR osteopathic OR massage OR masseuse OR passive jaw motion device OR continuous passive motion OR mulligan

3. physical therapy OR Exercise therapy OR exercise movement techniques OR exercise OR muscle stretching exercises OR weight lifting OR physical and rehabilitation medicine OR rehabilitation OR posture OR relaxation training OR yoga OR pilates OR resistance activity OR weight training OR resistance training OR strength training OR muscle strengthening OR weightlifting OR progressive resistance OR gravity resistive OR isotonic contraction OR isometric contraction OR eccentric OR concentric contraction OR training program OR training protocol OR training regime OR training strategy OR training therapy OR training intervention OR training progressive OR training functional OR training home OR training home-based OR training outpatient

4. ultrasound OR transcutaneous electrical stimulation OR electrostimulation OR interferential current OR biofeedback OR laser OR shortwave OR electromagnetic fields OR heat OR cold OR iontophoresis OR electrophoresis OR photo biomodulation OR phototherapy OR electroacupuncture OR burst modulated alternating current OR russian current OR microcurrents OR electrotherapy OR inferential therapy

5. electroacupuncture OR acupressure OR acupressure OR laser acupuncture OR auricular acupuncture OR acupuncture OR medical acupuncture

6. laser therapy OR low level laser therapy OR low intensity laser therapy OR low energy laser therapy OR infrared laser OR IR laser OR diode laser OR soft laser

7. transcutaneous electric nerve stimulation OR pulsed radio frequency energy OR diathermy

8. exp clinical trial OR randomized.tw. OR placebo OR randomly.tw. OR groups.tw.

9. neurobiofeedback OR brainwave biofeedback OR electromyography feedback OR biofeedback

10. cognitive behavioral therapy OR cognitive therapy OR acceptance and commitment therapy OR mindfulness OR behaviour therapy

11. #2 OR #3 OR #4 OR #5 OR #6 OR #7 OR #9 OR #10

12. #11 AND #8

13. #1 AND #12

14. #1 AND #11

- **<u>Ovid MEDLINE(R) ALL</u>**

Number of results: 109

1. exp tooth diseases/ or exp bruxism/ or sleep bruxism/
2. awake bruxism.mp.
3. 1 or 2
4. musculoskeletal manipulations/ or manipulation, chiropractic/ or manipulation, osteopathic/ or therapy, soft tissue/
5. orthopedic.mp.
6. exp musculoskeletal manipulations/ or exp manipulation, orthopedic/ or exp manipulation, osteopathic/ or manipulation, spinal/ or exp therapy, soft tissue/ or myofunctional therapy/
7. exp physical therapy modalities/ or exp dry needling/
8. manual therapy.mp.
9. exp Relaxation Therapy/
10. exp drainage/ or exp drainage, postural/ or exp manual lymphatic drainage/
11. exp spine/ or exp joints/
12. exp Back/

13. exp acupressure/ or exp massage/
14. exp Motion Therapy, Continuous Passive/
15. mulligan.mp.
16. 4 or 5 or 6 or 7 or 8 or 9 or 10 or 11 or 12 or 13 or 14 or 15
17. physical therapy modalities/ or exercise movement techniques/ or exercise therapy/ or hydrotherapy/ or exp rehabilitation/
18. exp exercise/ or exp muscle stretching exercises/ or exp running/ or exp swimming/ or exp walking/ or exp muscle strength/
19. exp weight lifting/ or exp relaxation/
20. (physical and rehabilitation medicine).mp. [mp=title, abstract, original title, name of substance word, subject heading word, floating sub-heading word, keyword heading word, organism supplementary concept word, protocol supplementary concept word, rare disease supplementary concept word, unique identifier, synonyms]
21. exp Posture/th [Therapy]
22. exp Yoga/th [Therapy]
23. pilates.mp.
24. resistance activity.mp.
25. weight training.mp.
26. exp endurance training/ or exp resistance training/
27. strength training.mp.
28. progressive resistance.mp.
29. gravity resistive.mp.
30. exp muscle contraction/ or exp isometric contraction/ or exp isotonic contraction/
31. eccentric contraction.mp.
32. concentric contraction.mp.
33. training program.mp.
34. training protocol.mp.
35. training regime.mp.
36. training strategy.mp.
37. training therapy.mp.
38. training intervention.mp.
39 training progressive.mp.
40. training functional.mp
41 training home.mp.

42. training outpatient.mp.
43. 17 or 18 or 19 or 20 or 21 or 22 or 23 or 24 or 25 or 26 or 27 or 28 or 29 or 30 or 31 or 32 or 33 or 34 or 35 or 36 or 37 or 38 or 39 or 40 or 41 or 42
44. ultrasound.mp.
45. exp electric stimulation therapy/ or exp transcutaneous electric nerve stimulation/ or exp laser therapy/ or exp phototherapy/
46. exp cryotherapy/ or exp electric stimulation therapy/ or exp electroacupuncture/ or exp pulsed radiofrequency treatment/
47. interferential current.mp.
48. exp short-wave therapy/ or exp ultrasonic therapy/
49. exp Electromagnetic Fields/st [Standards]
50. thermotherapy.mp.
51. exp iontophoresis/ or exp phonophoresis/
52. exp Electrophoresis/tu [Therapeutic Use]
53. photobiomodulation.mp.
54. burst-modulated alternating current.mp.
55. russian current.mp.
56. microcurrents.mp.
57. Electrotherapy.mp.
58. 44 or 45 or 46 or 47 or 48 or 49 or 50 or 51 or 52 or 53 or 54 or 55 or 56 or 57
59. exp acupuncture therapy/ or exp acupuncture analgesia/ or exp acupuncture, ear/ or exp moxibustion/ or exp auriculotherapy/ or exp cupping therapy/
60. laser acupuncture.mp.
61. 59 or 60
62. exp laser therapy/ or exp low-level light therapy/
63. low energy laser therapy.mp.
64. low intensity laser therapy.mp.
65. infrared laser.mp.
66. diode laser.mp.
67. soft laser.mp.
68. 62 or 63 or 64 or 65 or 66 or 67
69. exp Diathermy/tu, th [Therapeutic Use, Therapy]
70. exp biofeedback, psychology/ or exp neurofeedback/
71. brainwave biofeedback.mp.

72. electromyography biofeedback.mp.
73. 70 or 71 or 72 11388 Advanced More
74. exp cognitive behavioral therapy/ or exp "acceptance and commitment therapy"/ or exp mindfulness/
75. 16 or 43 or 58 or 61 or 68 or 69 or 73 or 74
76. 3 and 75
77. exp clinical trial/ or exp controlled clinical trial/
78. exp randomized controlled trial/
79. randomly.mp.
80. 77 or 78 or 79
81. 76 and 80

- **Embase**

Number of results: 88

1. (bruxism 'sleep bruxism' 'awake bruxism' manipulation orthopedic chiropractic 'spinal manipulation' 'spinal adjustment' 'musculoskeletal therapy' 'musculoskeletal manipulations' 'manual therapy' 'physical therapy modalities' 'relaxation therapy' 'orthopedic procedures' 'osteopathic medicine' 'drainage' 'dry needling' 'myofunctional therapy' spine spinal back osteopathic massage masseuse 'passive jaw motion device' 'continuous passive motion' mulligan ultrasound 'transcutaneous electrical stimulation' 'electrostimulation' 'interferential current' biofeedback laser shortwave 'electromagnetic fields' heat cold iontophoresis electrophoresis photobiomodulation phototherapy 'burst modulated alternating current' 'russian current' 'microcurrents' 'electrotherapy' 'interferential therapy' 'physical therapy' 'exercise therapy' 'exercise movement techniques' 'exercise' 'muscle stretching exercises' 'weight lifting' 'physical and rehabilitation medicine' 'rehabilitation' posture 'relaxation training' yoga pilates 'resistance activity' 'weight training' 'resistance training' 'strength training' 'muscle strengthening' 'weightlifting' 'progressive resistance' 'gravity resistive' 'isotonic contraction' 'isometric contraction' 'eccentric' 'concentric contraction' 'training program' 'training protocol' 'training regime' 'training strategy' 'training therapy' 'training intervention' 'training progressive' 'training functional' 'training home' 'training home-

based' 'training outpatient' electroacupuncture acupressure 'laser acupuncture' 'auricular acupuncture' 'acupuncture' 'medical acupuncture' 'laser therapy' 'low level laser therapy' 'low intensity laser therapy' 'low energy laser therapy' 'infrared laser' 'ir laser' 'diode laser' 'soft laser' 'transcutaneous electric nerve stimulation' 'pulsed radio frequency energy' 'diathermy' neurofeedback 'brainwave biofeedback' 'electromyography feedback' 'biofeedback' 'cognitive behavioral therapy' 'cognitive therapy' 'acceptance and commitment therapy' mindfulness 'behavior therapy' 'clinical trial' 'clinical trial' 'randomized' 'placebo' 'placebo' 'randomly' groups)
